# 965 Assessing the Implementation of Depression Screening in a Burn Clinic

**DOI:** 10.1093/jbcr/iraf019.496

**Published:** 2025-04-01

**Authors:** Morgan Colson, Lauren Baxley

**Affiliations:** Arkansas Children’s Burn Program; Arkansas Children’s Hospital

## Abstract

**Introduction:**

Burn injuries in pediatrics and adults are often traumatic experiences, resulting in depression. Burn patients are at higher risk for posttraumatic stress disorder, which can contribute to depression symptoms. The American Academy of Pediatrics recommends screening children aged 12 and older annually for depression, suggesting the Patient Health Questionnaire-9 (PHQ-9) is a reliable tool. In 2014, United States Preventive Services Task Force issued a recommendation to screen adults 18 and older for depression when support care is in place. The PHQ-9 tool has been utilized in standard care in this burn clinic since January 2023 for patients aged 12 and older. Prior to implementing this change, there were no standardized depression screenings in place. Adult patients had access to psychology one day per week, which may or may not have included a PHQ-9 screen. Pediatric patients had neither access to pediatric psychology nor standardized depression screenings. This is a quality improvement (QI) project to assess the implementation of a depression screening tool to determine total number of positive scores, positive scores in pediatric vs. adult patients, and need for future treatment and referrals for a positive score.

**Methods:**

In this clinic, patients 12 and older are screened with the PHQ-9 tool at initial clinic visits and re-screened in 6 months. PHQ-9 should not be used for patients < 12 years old or in patients with cognitive or developmental delay. The patient rates the nine questions on a Likert scale: “0-not at all,” “1-several days,” “2-more than half the days,” and “3-nearly every day.” The last question determines the functionality of the patient in daily life. The patient scores this question from “not difficult at all” to “extremely difficult.” The total PHQ-9 score is then compiled into the following categories of depression: Scores between 1-4: None; Scores 5-9: mild; Scores 10-14: moderate; Scores 15-19: moderate severe; and scores 20-27: severe. Total scores for this QI project were compiled through an Epic report.

**Results:**

Total number of patients screened with PHQ-9 from January 2023-January 2024 is 462. Fifty-three patients screened were 12-18 years old, with 21 patients resulting in positive scores. Four hundred-two patients screened were >18 years old, with 128 being positive.

**Conclusions:**

This QI project determined the rate of burn survivors endorsing symptoms of depression on the PHQ-9 scale from January 2023-January 2024. Upon conclusion, 149 out of 462 burn patients scored positive for depression, indicating possible need for further treatment or referrals.

**Applicability of Research to Practice:**

Final data will influence further QI projects in the burn clinic, including re-screening standards, treatment, and referrals for positive scores.

**Funding for the Study:**

No external funding utilized.

